# Association of Mucin-Degrading Gut Microbiota and Dietary Patterns with Colonic Transit Time in Constipation: A Secondary Analysis of a Randomized Clinical Trial

**DOI:** 10.3390/nu17010138

**Published:** 2024-12-31

**Authors:** Xuangao Wu, Hee-Jong Yang, Myeong-Seon Ryu, Su-Jin Jung, Kwangsu Ha, Do-Yeon Jeong, Sunmin Park

**Affiliations:** 1 Department of Bioconvergence, Hoseo University, 165 Sechul-Ri, BaeBang-Yup, Asan 31499, ChungNam-do, Republic of Korea; niyani0@naver.com; 2 Department of R&D, Microbial Institute for Fermentation Industry, 61-27 Minsokmaeul-gil, Sunchang-gun 56048, Republic of Korea; godfiltss@naver.com (H.-J.Y.); rms6223@naver.com (M.-S.R.); ksnova1492@naver.com (K.H.); 3 Research Institute of Clinical Medicine, Jeonbuk National University, Jeonju 54907, Republic of Korea; sjjeong@jbctc.org; 4 Department of Food and Nutrition, Obesity/Diabetes Research Center, Hoseo University, 165 Sechul-Ri, BaeBang-Yup, Asan 31499, ChungNam-do, Republic of Korea

**Keywords:** subjective constipation, colonic transit time, enterotypes, mucin-degrading bacteria, fermented beans

## Abstract

Background: The relationship between gut microbiota composition, lifestyles, and colonic transit time (CTT) remains poorly understood. This study investigated associations among gut microbiota profiles, diet, lifestyles, and CTT in individuals with subjective constipation. Methods: We conducted a secondary analysis of data from our randomized clinical trial, examining gut microbiota composition, CTT, and dietary intake in baseline and final assessments of 94 participants with subjective constipation. Participants were categorized into normal-transit (<36 h) and slow-transit (≥36 h) groups based on CTT at baseline. Gut microbiota composition was measured using 16S rRNA sequencing, and dietary patterns were assessed through semi-quantitative food frequency questionnaires. Enterotype analysis, machine learning approaches, and metabolic modeling were employed to investigate microbiota–diet interactions. The constipated participants primarily belonged to Lachnospiraceae (ET-L). Results: The slow-transit group showed higher alpha diversity than the normal-transit group. *Butyricicoccus faecihominis* was abundant in the normal-transit group, while *Neglectibacter timonensis*, *Intestinimonas massiliensis*, and *Intestinibacter bartlettii* were abundant in the slow-transit group, which also had a higher abundance of mucin-degrading bacteria. Metabolic modeling predicted increased N-acetyl-D-glucosamine (GlcNAc), a mucin-derived metabolite, in the slow-transit group. Network analysis identified two microbial co-abundance groups (CAG3 and CAG9) significantly associated with transit time and dietary patterns. Six mucin-degrading species showed differential correlations with GlcNAc and a plant-based diet, particularly, including rice, bread, fruits and vegetables, and fermented beans. In conclusion, an increased abundance of mucin-degrading bacteria and their predicted metabolic products were associated with delayed CTT. Conclusion: These findings suggest dietary modulation of these bacterial populations as a potential therapeutic strategy for constipation. Moreover, our results reveal a potential immunometabolic mechanism where mucin-degrading bacteria and their metabolic interactions may influence intestinal transit, mucosal barrier function, and immune response.

## 1. Introduction

Chronic constipation is a common gastrointestinal disorder affecting approximately 14–16% of the global population, with a higher prevalence among women and older adults. Although not life-threatening, chronic constipation significantly diminishes quality of life and is associated with an increased risk of severe health conditions, including colorectal cancer and cardiovascular diseases [1,2,3]. Clinically, it is a multifaceted condition characterized by the Rome IV diagnostic criteria, which include symptoms such as straining during defecation, hard stools, a sensation of anorectal blockage, and fewer than three spontaneous bowel movements per week, persisting for at least three months [4]. Objective assessments are critical for comprehensively understanding constipation, as subjective experiences alone may not align with measurable parameters. Radiopaque marker studies provide a quantitative evaluation of colonic transit time, with severe constipation typically defined as a transit time exceeding 36 h [5,6]. This metric offers valuable insights into gastrointestinal motility and the severity of the condition.

Emerging evidence highlights the gut microbiome as a key factor in understanding chronic constipation [7,8]. This complex ecosystem of microorganisms is vital to gastrointestinal and immune function and has been linked to neurological signaling via the gut–brain axis [9,10]. Dysbiosis, or microbial imbalance, has been implicated in various forms of constipation, underscoring the intricate relationships between gut microbiota, mucosal immunity, and gut–brain communication [11]. Altered gut–brain signaling can contribute to discomfort and the sensation of incomplete evacuation, even in individuals with normal bowel movement frequencies. Moreover, dietary factors, particularly fiber intake, play a critical role in modulating gut microbiota composition and bowel function to affect constipation, further complicating this interplay [12]. Together, these findings underscore the multifactorial nature of constipation, which extends beyond mechanical disruptions in transit.

This study investigated individuals who reported subjective symptoms of constipation while undergoing objective measurements of colonic transit time. This dual approach enabled us to explore the gut microbiota composition in a cohort with both self-reported and measurable markers of constipation. The gut–brain axis was a particular focus, as altered signaling within this system may drive the perception of constipation, even in the absence of objective motility issues [7,8,10]. To expand on prior research, we reanalyzed data from a randomized clinical trial examining the effects of chungkookjang (CKJ) on constipation. This reanalysis offered a unique opportunity to explore relationships among gut microbiota composition, colonic transit time, and subjective constipation experiences.

The objectives of our study were as follows: (1) to identify gut microbiota profiles associated with delayed colonic transit time (≥36 h) in participants with perceived constipation; (2) to evaluate the impact of dietary factors, including fiber intake and specific food groups, on gut microbiota composition and colonic transit time; and (3) to investigate interactions between gut microbiota, diet, and subjective constipation experiences, aiming to elucidate microbiota-related factors that may influence the perception of this condition. Through these objectives, this study seeks to enhance our understanding of the role of the gut microbiota in constipation. The findings may pave the way for personalized interventions targeting microbiota and dietary factors, offering improved management strategies for individuals affected by this burdensome condition.

## 2. Materials and Methods

### 2.1. Participants

This study was conducted with the approval of the Institutional Review Board of Jonbuk National University Hospital (CBNUH_CTCF2_IRB_2020-01-002). The study adhered to the principles of the Helsinki Declaration and the International Conference on Harmonization (ICH) Good Clinical Practice standards. Approval for secondary data usage was included in the IRB review. The study protocol was registered in the Korea Database Center for Medical Information (https://cris.nih.go.kr, accessed on 14 September 2021; KCT0005622), and the dietary intervention took place over eight weeks from 5 June 2020 to 15 March 2021. The sample size was calculated based on the assumption of a mean change in colonic transit time of −7.4\h for the CKB group and 1.9 h for the placebo group, with a standard deviation of 14.7 h, from the previous study [13]. To ensure 80% power at a 5% significance level, and accounting for a 20% dropout rate, 50 participants per group were required, for a total of 100 participants. From 116 volunteers who applied, 100 participants meeting the selection criteria were enrolled in the study after providing written informed consent.

### 2.2. Selection Criteria

Participants included in this study were men and women aged 19–40 years who met the Rome IV criteria for functional constipation, defined by symptoms such as straining during defecation, hard or lumpy stools, a sensation of anorectal blockage, and fewer than three spontaneous bowel movements per week [5,14]. Colonic transit time was not used as a selection criterion.

Participants were excluded if they had clinically significant allergies, gastrointestinal diseases that could be affected by the investigational product (CKJ), or a history of major gastrointestinal surgery excluding simple appendectomy or hernia repair. Additional exclusions included the use of gut-related medications or supplements within two months before screening, participation in other clinical studies within three months before screening, alcohol or drug abuse, or abnormal liver or kidney function tests. Individuals who were pregnant or breastfeeding, those unwilling to use contraception, and those with any other conditions deemed unsuitable for participation by the investigator were also excluded.

### 2.3. Study Design

**Figure 1:**
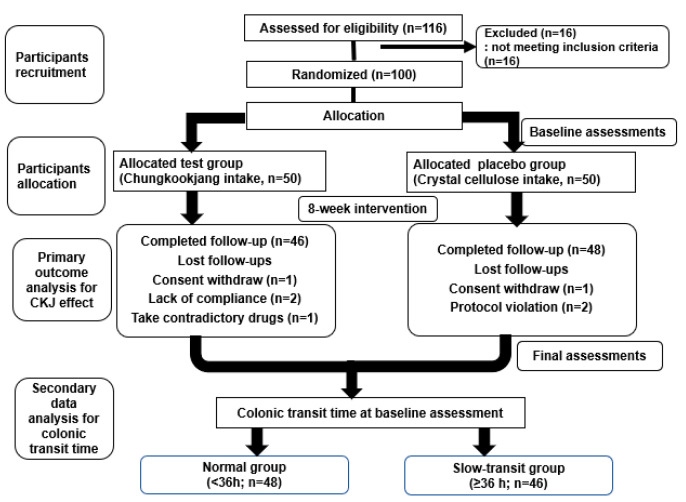
Study protocol for colonic transit time (CTT) assessment in intervention and control groups.

The flowchart of the randomized clinical trial and secondary data analysis is presented in Figure 1. This 8-week, randomized, controlled trial assessed the CKJ effects compared to a placebo in individuals with subjective constipation. The study included the following phases. (1) Screening period (from three weeks to four days before the study initiation): medical history, physical examination, and laboratory tests. (2) Baseline assessment (from three days before the study initiation up to Day 1): Colonic transit time was measured using radiopaque markers. (3) Randomization (Day 1): Participants were allocated in a 1:1 ratio to either a fermented soybean product (3000 mg CKJ plus 1.5 mg peanut powder per day) or a placebo (2976 mg crystal cellulose, 21 mg caramel color, and 4.5 mg peanut powder per day). (4) Intervention period: An 8-week daily supplementation of CKJ or crystal cellulose was given after breakfast. (5) Final assessment (54–57 days after study initiation): Colonic transit time measurements were conducted at both the baseline and final assessments.

### 2.4. Colonic Transit Time (CTT)

Colonic transit time (CTT) was measured using a multiple marker technique at baseline (Days −3 to 0) and at the end of the intervention period (Days 54–57). Participants ingested one capsule containing 20 radiopaque markers each day for three consecutive days. Abdominal X-rays were taken on Day 0 (the first visit) for the baseline measurement and Day 57 for the final assessment to count the number of markers remaining in the colon. CTT was calculated according to Metcalf’s method by multiplying the total number of remaining markers by 1.2 to express transit time in hours [13,15]. To ensure objective assessment, all X-ray readings were performed by trained radiologists who were blinded to the participants’ information using randomized identification codes. The blinding was maintained until study completion.

Normal CTT values vary according to age, gender, race, and methodology [6]. A threshold of 36 h is commonly used in clinical studies for constipation [16]. For the secondary analysis, participants were categorized into two groups based on their final colonic transit time: the normal-transit group with CTT < 36 h and the slow-transit group with CTT ≥ 36 h.

### 2.5. Clinical Measurements

Body weight and height were measured using a digital height and weight scale (G-Tech, Eujeongbu, Republic of Korea). Body mass index (BMI) was calculated by dividing body weight (kg) by height squared (m^2^). Bowel movement frequency, defecation time, the Bristol stool scale (BSS), and quality of life were assessed using questionnaires at baseline and final visits. Participants recorded the number of bowel movements per week and time spent during each defecation event in a daily diary. Quality of life was evaluated using the Patient Assessment of Constipation Quality of Life (PAC-QOL) questionnaire, which assessed constipation severity, impact on daily life, emotional changes due to constipation, and overall constipation-related life satisfaction. Patient Assessment of Constipation Symptoms (PAC-SYM) was also determined.

### 2.6. Biochemical Assays for Adverse Events and Safety Monitoring

Blood samples were collected at the baseline and final visits for biochemical analysis. The tests included white blood cell (WBC), red blood cell (RBC), and platelet count, hemoglobin, hematocrit, and the activities of γ-gamma-glutamyl transferase (GGT), aspartate aminotransferase (AST), and alanine aminotransferase (ALT), which indicate liver function. Indicators of kidney function (total bilirubin, total protein, blood urea nitrogen [BUN], and creatine kinase) were measured using a colorimetric method with a Hitachi 7600-110 (Hitachi, Tokyo, Japan). Albumin, total cholesterol, triglyceride, glucose, and high-sensitive C-reactive protein (hs-CRP) levels were evaluated in the blood. Urine tests were also performed. The blood and urine tests were conducted at the baseline and final assessments after eight weeks of intervention. Participants were trained to monitor and report any adverse events throughout the intervention periods. The vital signs, including systolic blood pressure, diastolic blood pressure, and pulse, were measured at each visit.

### 2.7. Dietary Intake

The dietary intake assessment was conducted using a 3-day food record method, a protocol previously validated in clinical trials at Jeonbuk National University Hospital. A trained dietician was instructed to record their dietary consumption comprehensively over three consecutive days, capturing typical eating patterns during the study period. Participants were assigned to consume tablets of either CKJ or placebo (microcrystal cellulose) in a double-blinded manner while maintaining their typical diet throughout the intervention period. Fermented foods were not allowed to be consumed during the intervention periods. The dietary intake analysis was performed using CAN-pro 4.0 (computer-aided nutritional analysis program, The Korean Nutrition Society Forum, Seoul, Republic of Korea) with data from the 56 days recorded during the eight-week study from which the average daily calorie and nutrient intakes were calculated.

Smoking history was assessed by documenting the duration of smoking (years) and the number of cigarettes smoked per day. Alcohol consumption was evaluated by recording drinking frequency, amount consumed per occasion, types of alcoholic beverages consumed, and calculating daily alcohol intake.

### 2.8. Acquisition of Fecal Bacteria Data of Healthy Persons

Preliminary analysis indicated that the 94 subjects with subjective constipation predominantly exhibited the Lachnospira enterotype. To confirm this finding, we compared the gut enterotypes of individuals with subjective constipation against those of healthy controls. Since the healthy control data lacked clinical information such as colonic transit time, they were used solely for enterotype classification purposes. Fecal samples were collected from the 94 participants with subjective constipation at baseline and at the end of the intervention.

As our study cohort did not include participants without subjective constipation, we supplemented our dataset with gut microbiome data from healthy Korean subjects without gastrointestinal diseases (including constipation), obtained from the European Nucleotide Archive (ENA, PRJEB33905) [17]. To ensure comparability, we randomly selected 190 samples from the 890 available healthy controls using Python’s random module (version 3.13.0), matching the sample size of our study cohort. This combined dataset allowed us to assess differences in gut enterotypes between individuals with subjective constipation and healthy controls.

### 2.9. Enterotype Analysis

To characterize the microbial community structure, we analyzed enterotypes in fecal samples of baseline and final assessment after the end of intervention for 94 participants following the methodology described by Arumugam et al. [18]. This analysis focused on genus-level clustering, excluding genera with average abundances below 0.1% to enhance analytical precision. The optimal number of enterotype clusters was determined using the Calinski–Harabasz (CH) index, and the cluster significance was evaluated using Chi-squared tests.

### 2.10. Gut Microbiome Analysis

The 16S rRNA sequencing data were analyzed using the Quantitative Insights into the Microbial Ecology Version 2 (QIIME2, version 2021.2) platform following standard bioinformatics procedures. The raw sequence data underwent initial demultiplexing using the Demux plugin to ensure sample-specific analysis. Quality control and denoising were performed using the Divisive Amplicon Denoising Algorithm, version 2 (DADA2) plugin, with sequences trimmed from positions 10 to 246 to retain high-quality reads. Taxonomic classification was conducted using the National Center for Biotechnology Information (NCBI) Basic Local Alignment Search Tool (BLAST+) against the NCBI 16S ribosomal RNA database, with a 97% similarity threshold for taxonomic assignment. Following annotation, features with identical taxonomic classifications were merged, and relative abundances were calculated across different taxonomic levels (kingdom to species).

### 2.11. Alpha- and Beta-Diversity and Linear Discriminant Analysis Effect Size (LEfSe) Analysis

Microbial diversity was assessed using both alpha- and beta-diversity metrics. Alpha diversity was quantified using Shannon’s diversity index, calculated from species-level relative abundance data. Statistical comparisons between the groups were performed using the Mann–Whitney U test. Beta-diversity was evaluated using Bray–Curtis dissimilarity matrices, computed using the R packages vegan and ade4. Inter-group differences in community composition were assessed using the Adonis function in the vegan package, allowing for a comprehensive comparison between study groups and enterotype classifications.

To identify the differentially abundant taxa between groups, we employed LEfSe analysis. Species-level abundance data were normalized and processed using the Mothur software. Taxa with linear discriminant analysis (LDA) scores greater than 2 and *p*-values less than 0.05 were considered significantly different between groups, providing insights into the specific microbial signatures associated with different clinical phenotypes.

### 2.12. Machine Learning Analysis and Metabolic Modeling

We developed an Extreme Gradient Boosting (XGBoost)-based classifier to distinguish between healthy and constipated individuals using species-level operational taxonomy unit (OTU) data. The model was implemented using Python’s XGBoost library (version 3.13.0) with hyperparameters optimized through randomized search (300 iterations, 3-fold cross-validation). Features were selected based on Mann–Whitney U tests (*p* < 0.05), and the dataset was split into training and testing sets (8:2 ratio). Model performance was evaluated through 10-fold cross-validation, receiver operating characteristic (ROC) curve analysis, and bootstrap-derived confidence intervals (1000 iterations, 95% confidence level) for accuracy, sensitivity, specificity, precision, and the F1 score. Shapley Additive Explanations (SHAP) analysis was employed to interpret feature contributions to model predictions.

### 2.13. Construction of Personalized Metabolic Models and Flux Analysis

To understand how gut microorganisms process nutrients and interact metabolically, we developed personalized metabolic models for each participant. These models are computational representations that simulate how microorganisms convert dietary components into various metabolic products. We used two established computational frameworks: AGORA2 (Assembly of Gut Organisms through Reconstruction and Analysis) [19] and the COBRA (COnstraint-Based Reconstruction and Analysis) toolbox (version 3.0) [20].

The modeling process consisted of several steps. First, we used AGORA2, which provides detailed metabolic models for individual bacterial strains commonly found in the human gut. Since our microbial abundance data was measured at the species level (rather than individual strains), we merged strain-level models into species-level models using the COBRA toolbox’s (version 3.0) create PanModels function. We then processed our microbial abundance data (OTU table) by converting it to relative abundances (proportions summing to 1) and matched it with available AGORA2 models, resulting in 347 species for analysis.

To account for dietary influences, we incorporated nutritional data representing typical Korean dietary patterns, obtained from the National Health and Nutrition Examination of Korea. The nutrient composition was calculated using Can Pro software (Korean Nutrition Society, Seoul, Republic of Korea). This dietary information served as input for understanding how different nutrients might affect microbial metabolism. We then created personalized metabolic models for each participant (190 models in total) by combining their gut microbiota profiles with the metabolic capabilities of each bacterial species. We then created personalized metabolic models for each participant (190 models in total) by combining their gut microbiota profiles with the metabolic capabilities of each bacterial species.

Finally, we used the COBRA toolbox’s initMgPipe function (version 3.0) to simulate metabolite production and absorption for each participant’s gut microbiota community. All computational analyses were performed using MATLAB R2021b with IBM CPLEX (12.10) as the mathematical solver for efficient calculations.

### 2.14. Microbial Network Analysis and Functional Prediction

For network analysis, we selected species-level OTUs with relative abundances >0.1% and processed the data using Mothur (version 1.48.0). Data normalization was performed using the sub.sample function (size = 1000), excluding samples with total counts below 1000. Sparse Correlations for Compositional Data (SparCC) analysis was conducted using default parameters to account for the compositional nature of microbiome data.

Microbial co-abundance group (CAG) networks were constructed using Ward clustering (ward.D2 method) based on the SparCC correlation matrix. We first calculated the Euclidean distance of the correlation matrix, then performed hierarchical clustering using the ward.D2 method, and finally divided the results into 10 groups using the Cutree function. The relative abundances of bacteria belonging to the same CAG were summed to obtain the total relative abundance of each CAG.

Correlations among the dietary data, CAGs, species-level bacteria, and predicted metabolites were assessed using Pearson’s correlation analysis (Python Pandas package, version 3.13.0), with significance determined using the Scipy package. Network visualization was performed using Cytoscape 3.4.0, with nodes and edges colored to distinguish data types and correlation directions.

### 2.15. Prediction of Strains with Mucin Utilization Ability

We downloaded complete reference sequences of strains for the CAG3 and CAG9 groups from NCBI’s nucleotide database. We predicted the polysaccharide substrates that each strain could utilize using the Database for Automated Carbohydrate-active Enzyme Annotation (DBCAN) tool. By employing the run_dbcan function in DBCAN, we annotated the enzyme gene clusters (CGCs) in bacterial sequences, which were used to predict the corresponding usable CGC substrates by adding the cgc_substrate annotation in the command line [21]. Based on the results, we determined whether the bacteria could utilize mucin as a polysaccharide substrate and the number of related gene clusters they contained.

### 2.16. Statistical Analysis

Differences in the gut microbiota composition between the normal-transit and slow-transit groups were analyzed. Beta-diversity differences in the gut microbiota composition between the above slow-transit groups were evaluated using permutational multivariate analysis of variance (PERMANOVA). Individual taxa comparisons were performed using Mann–Whitney U tests, with data expressed as median ± interquartile range (IQR). Associations between microbial taxa, dietary factors, clinical measurements, and biochemical parameters were examined using Spearman’s rank correlation coefficient and multiple regression models, with CKJ consumption included as a covariate.

Constipation-related biomarkers and biochemical parameters between normal- and slow-transit groups were compared between the normal-transit and slow-transit groups using Analysis of Covariance (ANCOVA) with adjustment for multiple covariates including age, gender, daily energy intake, smoking duration, alcohol consumption, pre-existing medical conditions, CKJ intake, physical activity level, and dietary fiber intake. Statistical analyses were performed using the SAS (version 9.4; Cary, NC, USA) and R software (version 4.4.2), and the significance of differences was set at *p* < 0.05. For microbiome analyses, the false discovery rate (FDR) correction was applied to account for multiple comparisons.

## 3. Results

### 3.1. Chanracteistics of Participants

The normal- and slow-transit groups included more women than men, but the female percentage did not differ between the groups (Table 1). Age, BMI, lipid profiles, fasting glucose, liver damage index, and immune-related factors in the blood did not differ between the normal- and slow-transit groups (Table 1). Interestingly, the estimated values for serum triglyceride and glucose concentrations were significantly and inversely associated with colonic transit time, whereas serum platelet and creatinine levels were positively associated in the linear regression analysis (Table 1). During the study, six participants were lost due to consent withdrawal (*n* = 2), lack of compliance (*n =* 2), protocol violation (*n =* 1), and taking contraindicated drugs (*n =* 1). A total of 94 participants were included in the baseline and final assessments.

### 3.2. Food and Nutrient Intake and Lifestyles

The participants were categorized into three dietary patterns, comprising a healthy diet including grains, fish, and potatoes; a plant-based diet including sweetened bread, fruits, nuts, vegetables, and mushrooms; and a traditional Korean diet, including rice, soup, and kimchi. The percentages of participants in three dietary patterns did not significantly differ in baseline and final assessments (Table 2). Energy intake did not differ between the normal- and slow-transit groups in each session, but all participants had a low energy intake of about 61% of the estimated energy requirement (Table 2). Macronutrient intake was about 54 energy percent (En%), 16 En%, and 29 En% of carbohydrates, protein, and fat, respectively, which was the recommended proportion. Dietary fiber intake was about 15 g/day and showed no significant difference among the groups in both assessments (Table 2). Foods related to constipation, such as kimchi, jang, beans, fruits, mushrooms, and vegetables, did not differ between the normal- and slow-transit groups in each assessment (Table 2).

The number of cigarettes smoked per day showed no significant difference between the groups in each assessment, although it appeared to be higher in the normal-transit group than in the slow-transit group. Alcohol intake showed no significant difference between the normal- and slow-transit groups in both assessments.

### 3.3. Colonic Transit Time and Constipation-Related Parameters

**Figure 2:**
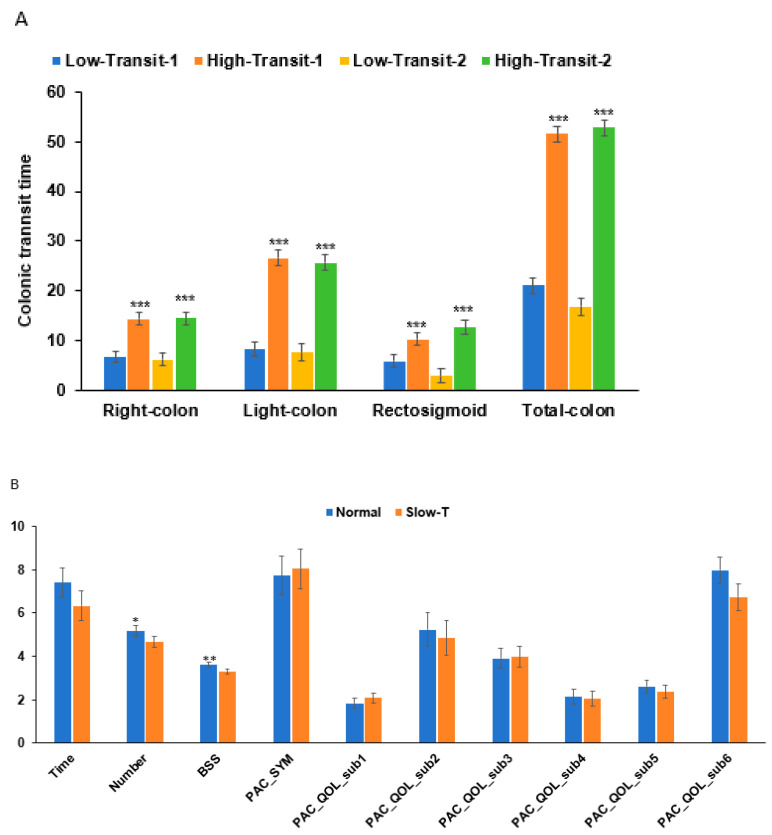
Colonic transit time and constipation symptoms. Colonic transit time between normal- and slow-transit (Slow-T) groups at the baseline and final assessments after the intervention. (**A**) Colonic transit time for different parts of the colon. (**B**): Constipation-related parameters between the Normal and Slow-T groups. The -1 and -2 in the name of the legends indicated baseline and final sessions. Normal- and slow-transit groups were compared using Analysis of Covariance (ANCOVA) with adjustment for multiple covariates, including age, gender, daily energy intake, smoking duration, alcohol consumption, pre-existing medical conditions, CKJ intake, physical activity level, and dietary fiber intake. Time, defecation time (min/each); Number, number of defecations per week; BSS, Bristol stool scale; PAC-SYM, patient assessment of constipation symptoms; PAC-QOL, patient assessment of constipation quality of life; PAC sub-categories: sub1, constipation intensity; sub2, daily life influence; sub3 and sub4, emotional changes due to constipation; sub5, daily health condition accompanied with constipation; sub6, satisfaction for improvement in constipation. Significant difference between the Normal and Slow-T groups at * *p* < 0.05, ** at *p* < 0.01, and *** at *p* < 0.001.

The transit times in passing the right, left, and rectosigmoid colon were much shorter in the normal-transit group than in the slow-transit group at baseline and final assessments. The interventions of CKJ and placebo did not alter the transit time. Although the colonic transit time significantly differed between the normal- and slow-transit time groups, the total colon transit time in the normal- and slow-transit groups was 21.1 and 51.5 h at baseline and 16.8 and 52.7 h in the final assessment after CKJ or placebo treatments (*p* < 0.001; Figure 2A). This indicated that the participants in the normal-transit group seemed to have lower transit times, but the difference was not significant.

The number of bowel movements and the time required to defecate were 9.19 and 7.59 times per week and 7.53 and 6.57 min per each time, respectively, in the normal- and slow-transit time groups at the final assessments after CKJ or placebo treatments (Figure 2B). The Bristol stool state (BSS) was 3.5 for the normal-transit group and 3 for the slow-transit group (*p* = 0.0017). The patient assessment of constipation symptoms (PAC-SYM) also did not differ between the normal- and slow-transit groups. The patient assessment of the constipation quality of life (PAC-QQL) subitems including constipation intensity (sub1), daily life influence (sub2), emotional changes due to constipation (sub3 and sub4), daily health condition accompanied with constipation (sub5), and satisfaction for improvement in constipation (sub6) showed no significant difference between the normal- and slow-transit groups (Figure 2B). These bowel-movement-related parameters appeared to be lower in the normal-transit group than in the slow-transit group. However, no significant differences were noted (Figure 2B). Therefore, the colonic transit time did not reflect the patient’s symptoms and quality of life.

### 3.4. Biochemical Parameters at the Final Assessment After CKJ Intervention

The biochemical markers related to adverse effects revealed no significant differences between the CKJ and placebo groups (Supplementary Table S1). Specifically, WBC, RBC, platelet count, AST, ALT, γ-GGT, and BUN concentrations were comparable between the two groups, indicating that the CKJ intervention did not have any adverse effects on these markers in either the normal- or slow-transit group. These results suggest that the CKJ intervention was safe and did not negatively impact hematological or liver function parameters, reinforcing that any observed effects in the primary outcomes related to normal vs. slow transit time were not confounded by biochemical safety concerns.

### 3.5. Analysis of Enterotypes in Healthy Individuals and Those with Subjective Constipation

**Figure 3:**
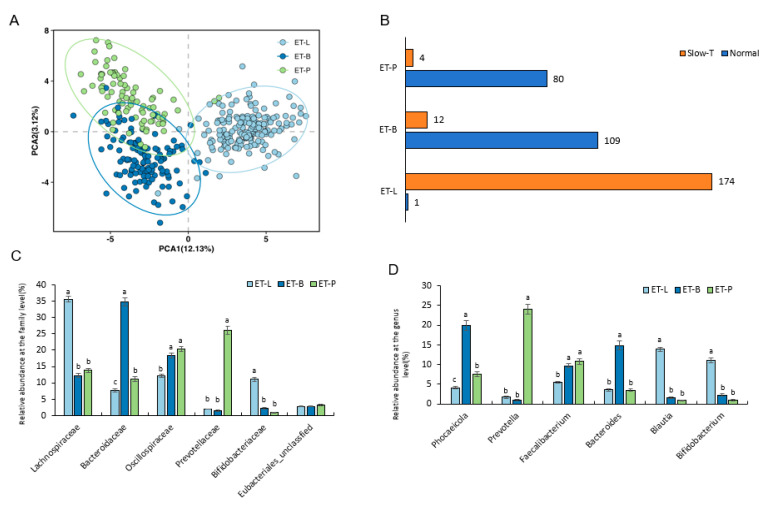
Characteristics and distribution of the three enterotypes from the subjective participants of the present study and the healthy subjects without gastrointestinal diseases of previous studies. Subjective constipation; Healthy control (Normal); ET-B, Bacteroidaceae enterotype; ET-L, Lachnospiraceae enterotype; ET-P, Prevotella enterotype. (**A**). Principal component analysis (PCA) plot of the three enterotypes based on fecal microbiota at the genus level. (**B**). The number of HC and CC subjects in each enterotype. The Chi-square test was used to statistically assess the significant differences in the number of subjects across the enterotypes. Slow-T, slow-transit group. (**C**). Relative abundance of the top six family-level taxa in each enterotype. Tukey’s post hoc test was employed to determine significant differences between enterotypes. Different letters on the bars indicated significant differences among the enterotypes at *p* < 0.05. (**D**). Relative abundance of the top six genus-level taxa in each enterotype. Tukey’s post hoc test was used to identify significant differences between enterotypes. a–c Different letters on the bars indicated significant differences among the enterotypes at *p* < 0.05.

To analyze the differences in enterotypes between individuals with subjective feelings of constipation and the healthy control group, we performed enterotype clustering using the relative abundance table at the genus level. A total of three enterotypes emerged, categorized by the relative abundance at the family level: the Bacteroidaceae enterotype (ET-B), Lachnospiraceae enterotype (ET-L), and the Prevotella enterotype (ET-P) (Figure 3A). The gut bacteria compositions of ET-B, ET-L, and ET-P are shown in Figure 3B,C. In the normal group, most individuals were ET-B and ET-P, with 109 and 80 people, respectively, while only 1 person was ET-L. In contrast, in the participants with subjective constipation, most individuals (*n* = 174) were ET-L, while 12 and 4 people were ET-B and ET-P, respectively (Figure 3D). The distribution of individuals across the enterotypes in the healthy and participants with subjective constipation, thus, showed significant differences (Figure 3D, *p* < 0.05).

### 3.6. Downstream Analysis of Gut Microbiota

**Figure 4:**
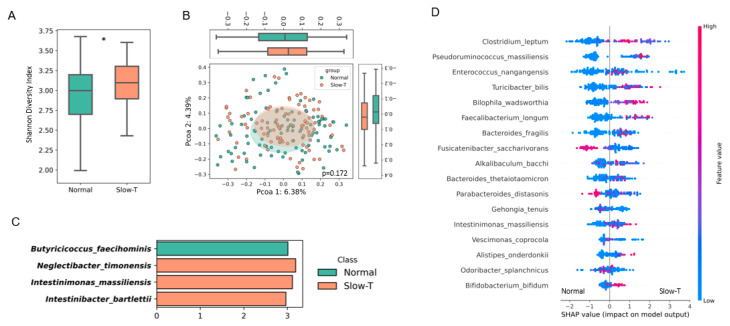
Gut microbiome analysis. (**A**). α-diversity Shannon’s diversity index in participants. (**B**). β-diversity in participants. (**C**). LEfSe analysis showing differential gut bacteria species. (**D**). SHAP summary plot for species-level bacterial features. The order of features represents their importance, and each point represents the SHAP value for a specific microbial species. The color indicates the effect of the feature value on the corresponding classification (blue represents low and red represents high). Groups were normal-transit (Normal; <36 h colonic transit time) and slow-transit (≥36 h colonic transit time; Slow-T), based on the colonic transit time test set data. SHAP, SHapley Additive exPlanations. * Significant difference between the Normal and Slow-T groups at *p* < 0.05.

The α-diversity results showed that the Shannon index in the slow-transit group was significantly higher than that in the normal-transit group (Figure 4A, *p* < 0.05). However, the β-diversity analysis revealed no significant differences between the normal- and slow-transit groups (Figure 4B, *p* > 0.05). The LefSe analysis, using a univariate framework, revealed that the normal-transit group was significantly enriched with *Butyricicoccus faecihominis*, while the slow-transit group had significantly higher abundances of *Neglectibacter timonensis*, *Intestinimonas massiliensis*, and *Intestinibacter bartlettii* (Figure 4C, *p* < 0.05). In contrast, the XGBoost classification model, which considered multivariate interactions, achieved an AUC value of 0.606 in distinguishing between the normal- and slow-transit groups. SHAP analysis was used to rank the importance of the top 20 bacterial species in the model. In the slow-transit group, species such as *Clostridium leptum*, *Pseudoruminococcus massiliensis*, *Turicibacter billis*, *Intestinimonas massiliensis*, and *Biophilia wadsworthia* were more abundant compared to the normal-transit group (Figure 4D). Higher SHAP values indicated a greater risk of being classified in the slow-transit group, with red dots representing higher feature values and blue dots representing lower values. SHAP values on the x-axis provided a unified measure of each feature’s influence on the model’s predictions (Figure 4D). The differences in the bacteria identified by LefSe and SHAP analyses reflect their distinct statistical approaches. While LefSe highlights bacteria based on univariate significance, the XGBoost-SHAP framework captures complex, multivariate interactions. Combining these approaches enhanced the identification of potential microbial biomarkers and therapeutic targets for gastrointestinal motility disorders.

**Figure 5:**
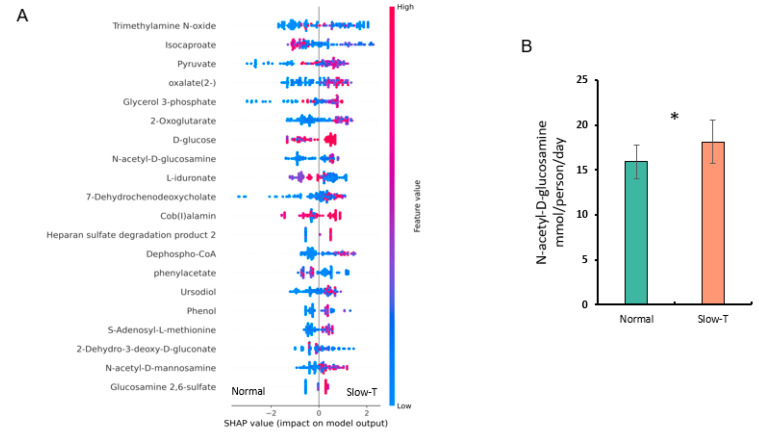
Analysis of the predicted metabolic features of the gut microbiome using the COBRA toolbox and AGORA2 model. (**A**). SHAP summary plot for predicted microbial metabolic feature data. The order of features represents their importance, and each point represents the SHAP value for a specific microbial species. The color indicates the effect of the feature value on the corresponding classification (blue represents low and red represents high). (**B**). Bar chart of N-acetyl-D-glucosamine levels between groups. * Significant differences between the groups at *p* < 0.05. Groups were normal-transit (Normal; <36 h colonic transit time) and slow-transit (≥36 h colonic transit time; HT), based on the colonic transit time test set data. SHAP, SHapley Additive exPlanations.

In an XGBoost classification model for the microbial metabolites predicted by the COBRA toolbox and the AGORA2 model, the top 20 important metabolic features were ranked using SHAP (Figure 5A). Among the significant metabolic features, we found that the mucin-derived metabolite N-acetyl-D-glucosamine was notably increased in the slow-transit group (Figure 5B, *p* < 0.05).

### 3.7. Multifactorial Network Analysis

**Figure 6:**
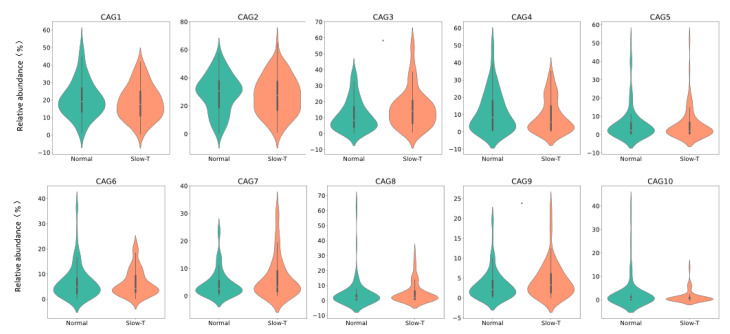
Violin plot of the relative abundance of species in co-abundance groups (CAGs). The total relative abundance of each CAG was calculated by summing the relative abundances of species belonging to the same CAG.

Using SparCC and Ward’s minimum variance method, we clustered the species into 10 clusters. Among these clusters, CAG3 and CAG9 were significantly increased in the slow-transit group, suggesting their important roles in distinguishing between the normal- and slow-transit groups (Figure 6, *p* < 0.05).

**Figure 7:**
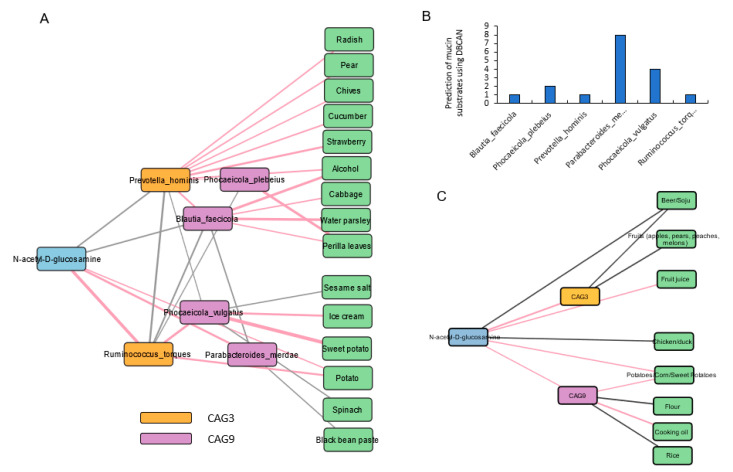
Correlation network analysis between N-acetyl-D-glucosamine, co-abundance groups (CAG) clusters, species strains, and diet. (**A**). Correlation analysis of mucin-utilizing species in CAG3 and CAG9 with N-acetyl-D-glucosamine and diet. (**B**). DBCAN analysis of mucin metabolism substrate utilization in species within CAG3 and CAG9. (**C**). Correlation analysis between N-acetyl-D-glucosamine, CAG clusters, and diet.

To further investigate the relationships between diet, gut microbiota, and N-acetyl-D-glucosamine, we performed Pearson’s correlation analysis and visualized the network. Both CAG3 and CAG9 showed significant positive correlations with N-acetyl-D-glucosamine, with CAG3 displaying a higher correlation than CAG9. Beer/soju and fruits (apples, pears, peaches, and melons) showed significant negative correlations with CAG3, while fruit juice had positive correlations with CAG3. Potatoes/corn/sweet potatoes and cooking oil intakes were positively associated with CAG9 and N-acetyl-D-glucosamine, whereas flour and rice had negative correlations with CAG9 (Figure 7A, *p* < 0.05). Since N-acetyl-D-glucosamine is a bacterial metabolite of mucin, six mucin-degrading bacteria species were identified to predict the mucin-degrading species within CAG3 and CAG9 using DBCAN analysis (Figure 7B). The six species were classified based on the positive and negative correlations with N-acetyl-D-glucosamine by Pearson’s correlation analysis (Figure 7C). We found that *Parabacteroides merdae* and *Ruminococcus torques* were significantly positively correlated with N-acetyl-D-glucosamine, and *Phocaeicola vulgatus* also showed a positive correlation. Conversely, *Prevotella hominis* and *Blautia faecicola* showed significant negative correlations with N-acetyl-D-glucosamine. Concerning the diet, beer/soju was positively correlated with *Blautia faecicola*, while fruits (pears, strawberry) and vegetables (radish, chives, cucumber, cabbage, and perilla leaves) were positively correlated with *Prevotella hominis*, *Phocaeicola plebeius*, and *Blautia faecis*, which had a lower mucin degrading scores (Figure 7C, *p* < 0.05). They were inversely related to N-acetyl-D-glucosamine contents. In contrast, the three bacteria (*Parabacteroides merdae*, *Phocaeicola vulgatus*, and *Ruminococcus torques*) having higher mucin degrading scores were positively correlated with N-acetyl-D-glucosamine and they were positively correlated with ice cream, sweet potato, and potato intakes and inversely correlated with sesame salt, spinach, and black bean paste (fermented soybeans) intakes. The results suggested that some fruits and vegetables, but not fruit juice, sweet potato, and potato, were positively associated with mucin-degrading bacteria and inversely associated with bacteria such as *Prevotella hominis*, *Phocaeicola plebeius*, *Blautia faecicola*, and *Phocaeicola vulgatus*, which may modulate functional constipation.

## 4. Discussion

In this study, we investigated the relationship between gut microbiota composition, dietary patterns, and colonic transit time in individuals with subjective constipation. Our findings reveal several novel insights: (1) all participants with subjective constipation predominantly belonged to the Lachnospiraceae enterotype (ET-L) compared with individuals without subjective symptoms of constipation; (2) the increased abundance of mucin-degrading bacteria, particularly in those with CAG3 and CAG9, was positively associated with delayed transit time; and (3) specific dietary components showed significant positive correlations with mucin-degrading bacteria abundance and transit time. The predominance of ET-L in individuals with subjective constipation, regardless of colonic transit time, represents a significant departure from the microbiota profiles typically observed in healthy individuals. Previous studies have reported that gut microbiota in Koreans without gastrointestinal diseases is generally dominated by either the Bacteroidaceae (ET-B) or Prevotella (ET-P) enterotypes [17]. However, studies of healthy or overweight Koreans have identified three distinct enterotypes: ET-B, ET-P, and ET-L [22]. Our findings of ET-L dominance in individuals with subjective constipation align with recent work [23,24] demonstrating increased Lachnospiraceae abundance and decreased Bacteroidetes and *Prevotella* in functional constipation. Additionally, a higher number of Asians with depression belong to ET-L than ET-B and ET-P [25]. However, our study extends these findings by demonstrating the specific association between ET-L and the perception of constipation, regardless of colonic transit time, through the alteration of the gut–brain axis.

The discordance between subjective constipation and objective constipation (defined by colonic transit time) observed in our study can be explained by various mechanisms involving the gut–brain axis. Previous studies have shown that constipation involves increased sympathetic and reduced parasympathetic nervous system activity, indicating disturbed gut–brain communication [26]. This relationship is particularly evident in neurological conditions such as Parkinson’s disease and multiple sclerosis, where objective dysfunction often exceeds subjective symptoms despite significant delays in colonic transit time [26,27]. Several mechanisms may explain this paradox, aligning with our findings that participants with subjective symptoms of constipation had either normal or slow transit times. Visceral hypersensitivity and rectal hyposensitivity can alter the perception of normal bowel function through modified sensory processing [28,29]. These alterations stem from impaired afferent nerve function or changes in central processing of visceral sensations [30]. Additionally, pelvic floor dysfunction, characterized by paradoxical contraction or inadequate relaxation, can create constipation symptoms regardless of transit time [31]. Psychological factors, particularly anxiety and somatization, can amplify the perception of gastrointestinal symptoms independent of actual transit time [32]. Our findings of altered microbiota composition complement previous research showing that mucosal inflammation and modified gut microbiota can influence both motility and sensory function [33,34]. These microbiota-related changes could also influence mucosal immune responses and gut barrier function, potentially contributing to the perception of constipation symptoms. This suggests that the microbiota–gut–brain axis plays a crucial role in manifesting constipation symptoms, potentially explaining the variable relationship between subjective symptoms and objective measures of constipation observed in our study.

Our findings demonstrated that individuals with subjective constipation, regardless of their colonic transit time, shared a common ET-L (specifically *Lachnospira*). This finding is significant as it suggests that perception of constipation might be more closely linked to disruption of the gut–brain axis than mechanical transit issues, and is related to involvement of Lachenospiraceae in neural signaling through the vagal pathways [35]. Notably, Lachenospiraceae has been associated with various neurological conditions, including major depressive disorder and multiple sclerosis [25,35,36,37] with the vagus nerve mediating gut–brain communication to modulate constipation [12,38].

Our study revealed a significant positive correlation between mucin-degrading bacteria and delayed colonic transit time, evidenced by increased N-acetyl-D-glucosamine (GlcNAc) produced by mucin-metabolizing gut microbiota in patients with constipation. GlcNAc is a component of intestinal mucin widespread in gut oligosaccharides with a core 3 structure, namely, GlcNAc(β1-3)GalNAc [39]. The mucin layer, a highly glycosylated protein layer, acts as a lubricant for the passage of food, participates in cell signaling, and protects the host epithelium from pathogens and environmental toxins [40]. Studies show decreased soluble mucin secretion in patients with constipation compared to healthy volunteers [41]. Mucin-degrading bacteria and their metabolites, such as GlcNAc, are potential biomarkers for constipation and potential targets for prevention and treatment interventions. While our findings suggest GlcNAc’s association with constipation based on computational predictions, further experimental validation is necessary to confirm its clinical utility. Future research should focus on directly measuring GlcNAc levels, establishing reference ranges, and validating these findings in larger, prospective studies. These efforts will be critical for developing reliable, clinically applicable biomarkers for diagnosing and managing constipation.

In our clustering analysis, CAG3 and CAG9, which were elevated in patients with subjective symptoms of constipation, showed a positive correlation with GlcNAc production. The mucin-degrading bacteria within these CAGs displayed two distinct patterns: one group was positively correlated with GlcNAc production, while the other showed a negative correlation. We identified three bacterial species (*Parabacteroides merdae*, *Ruminococcus torques*, and *Phocaeicola vulgatus*) with enhanced mucin-degrading abilities significantly associated with transit time. Mucin, continuously secreted by the intestinal goblet cells, provides energy for the survival of colonic bacteria [42]. During the utilization of mucin by the bacteria, goblet cells are stimulated to secrete more mucin, with GlcNAc inducing mucin secretion from the colonic tissue [43]. However, dysbiosis, pathogenic overgrowth, and excessive mucin consumption can disrupt the intestinal barrier system [42]. The relationship between mucin-degrading bacteria and delayed transit time operates through several mechanisms. These bacteria can metabolize the protective mucus layer, potentially altering gut barrier function and motility patterns [42], and regulate intestinal motility through metabolic products affecting the neural signaling pathways and smooth muscle contractility [44,45]. This creates a self-reinforcing cycle, where slower transit time allows increased bacterial colonization and mucus degradation [46].

Our study revealed distinct correlations between dietary components and mucin-degrading bacteria, extending beyond previous findings that showed the impact of a general diet on the gut microbiota [22,47]. Beer/soju and fruits showed negative correlations with CAG3, while cooking oil and yogurt demonstrated positive correlations with CAG9. Notably, we identified differential relationships between bacterial species and N-acetyl-D-glucosamine production: three species (*Parabacteroides merdae, Ruminococcus torques*, and *Phocaeicola vulgatus*) showed positive correlations and possessed more functional gene regions for mucin degradation, while *Prevotella hominis* and *Blautia faecicola* showed negative correlations with fewer genes for mucin degradation [48].

An intriguing finding was the paradoxical relationship between fruit consumption and bacteria in CAG3. While it positively correlated with specific mucin-degrading bacteria (*Phocaeicola vulgatus* and *Ruminococcus torques*), it showed a negative correlation with CAG3, suggesting complex interactions between dietary fiber and bacterial metabolism [49]. Additionally, our novel finding of the positive correlation of cooking oil with CAG9 builds upon previous research on the impact of dietary lipids on gut microbiota [50], providing specific insights into their relationship with mucin-degrading bacterial communities.

The primary strength of our study lies in its comprehensive methodological approach, integrating multiple analytical techniques, including enterotype analysis, machine learning, and metabolic modeling. This multi-faceted approach, with detailed dietary information, provided a robust framework for understanding the complex interactions between gut microbiota, diet, and transit time in constipation. Our study revealed several novel findings on the pathophysiology of constipation. We identified a distinct enterotype distribution pattern characterized by ET-L predominance in individuals with subjective constipation and discovered previously unreported associations between specific bacterial species and transit time. Two distinct microbial co-abundance groups (CAG3 and CAG9) had significant relationships with both transit time and dietary patterns. A key strength of the study was the identification of N-acetyl-D-glucosamine as a potential biomarker for delayed transit time and the characterization of specific mucin-degrading species and their correlations with dietary components and metabolites.

The primary limitation of this study is its case-control design, which restricts the ability to establish causal relationships between gut microbiota changes and colonic transit time. Additionally, the modest sample size (*n* = 94) limits the generalizability of the findings, highlighting the need for larger, more diverse cohorts and longitudinal studies to validate the results. Methodologically, while 16S rRNA sequencing provides robust taxonomic data, its resolution is lower than shotgun metagenomic sequencing, although functional predictions were supplemented using Picrust2. The 3-day food record was not cross-validated with other dietary assessment methods while routinely used for over 20 years in clinical trials at this facility, consistently providing reliable results for assessing food intake [13]. Furthermore, the binary categorization of colonic transit time may oversimplify its clinical variability. Finally, while the study identified significant associations between mucin-degrading bacteria and transit time, the underlying mechanistic pathways remain unclear and warrant further exploration in future research.

Our findings have several important clinical implications for the management of constipation. Identifying specific enterotype patterns and mucin-degrading bacterial populations provides new potential therapeutic targets for constipation treatment. The associations observed between dietary components and specific bacterial communities suggest that targeted dietary modifications, particularly for fruit consumption and fermented foods, could be implemented as part of personalized treatment strategies. Furthermore, the relationship between mucin-degrading bacteria, N-acetyl-D-glucosamine, and transit time suggests its potential use as a biomarker for monitoring treatment response in patients with constipation. These findings may help clinicians develop more effective, personalized treatment strategies that leverage dietary modifications and targeted microbiota-based interventions to address both the objective and subjective aspects of constipation.

## 5. Conclusions and Future Directions

Our comprehensive analysis reveals significant associations between gut microbiota composition, metabolic functions, and colonic transit time in individuals with subjective symptoms of constipation. Identifying distinct enterotypes and the specific bacterial species associated with transit time provides new insights into the microbial ecology of constipation. This connection exhibits potential interactions between microbial activity, nutritional status, and immune system responses. The complex interactions observed between dietary patterns and microbial communities, especially concerning mucin-degrading species and their metabolites, point to potential therapeutic interventions through dietary modulation.

Future research should extend these findings through longitudinal studies to establish causal relationships between the bacterial species identified in this study and colonic transit time. The development of targeted interventions, whether through specific dietary modifications or novel prebiotics and probiotics, warrants investigation. Further exploration of N-acetyl-D-glucosamine metabolism and its role in colonic transit could reveal new therapeutic targets. Additionally, validating these findings in larger, more diverse populations will be essential for developing personalized treatment strategies. Understanding the temporal dynamics of microbiota–diet interactions and their impact on transit time could lead to more effective, individualized approaches for managing constipation. These insights open new avenues for therapeutic interventions that could significantly improve the treatment of constipation through microbiota-based approaches. 

## Figures and Tables

**Table 1:** General characteristics of the participants in the baseline assessment.

	Normal (*n* = 48)	Slow-Transit (*n* = 46)	Regression Coefficient (β) for Colonic Transit Time
Age (years)	37.5 ± 1.32	37.2 ± 1.35	−0.0294 ± 0.028
Gender (Women, N, %)	40 (80.0)	44 (91.7)	0.0017 ± 0.001
BMI (kg/m^2^)	22.8 ± 0.41	22.2 ± 0.42	−0.0103 ± 0.010
Serum total cholesterol (mg/dL)	187 ± 6.2	182 ± 6.3	0.0715 ± 0.134
Serum triglyceride (mg/dL)	93.6 ± 6.39	84.9 ± 6.53	−0.380 ± 0.153 ^+^
Serum glucose	84.8 ± 0.94	81.7 ± 0.97	−0.0672 ± 0.0294 ^+^
Serum AST (IU/L)	22.3 ± 0.91	21.5 ± 0.92	−0.0161 ± 0.0211
Serum ALT (IU/L)	19.5 ± 1.09	18.2 ± 1.11	−0.0234 ± 0.0271
Serum ALP (IU/L)	54.3 ± 2.03	57.1 ± 2.07	0.0512 ± 0.0518
Serum BUN (mg/dL)	13.6 ± 2.71	13.2 ± 3.99	0.0197 ± 0.0124
Serum creatinine (mg/dL)	0.68 ± 0.01	0.72 ± 0.02	0.0012 ± 0.0004 ^++^
Plasma platelet (×10^11^/U)	251 ± 6.74	261 ± 6.88	0.4615 ± 0.1744 ^++^
Blood WBC (×10^9^/L)	5.10 ± 0.18	5.52 ± 0.18	0.0040 ± 0.0044
Exercise (min/week)	1353 ± 1202	1276 ± 1148	−0.0001 ± 0.0022
Current smoking (Yes, N, %)	7(14.0)	2 (4.17)	−0.0014 ± 0.0009
Smoking duration (year)	1.3 ± 0.5	0.4 ± 0.51	−0.0251 ± 0.013
Alcohol drinking duration (year)	7.43 ± 1.10	6.68 ± 1.12	−0.0067 ± 0.011

Values represent mean ± standard error or number (%). BMI, body mass index; AST, aspartate aminotransferase; ALT, alanine aminotransferase; ALP, alkaline phosphatase; BUN, blood urinary nitrogen; WBC, white blood cells. ^+^ Significant association between biochemical parameters and colonic transit time at *p* < 0.05, ^++^ at *p* < 0.01.

**Table 2:** Food and nutrient intake and lifestyles.

	Baseline Assessment		Final Assessment After Intervention	
	Normal (*n* = 48)	Slow-Transit (*n* = 46)	Normal (*n* = 48)	Slow-Transit (*n* = 46)
Healthy diet pattern	10 (21.7)	12 (26.1)	9 (20.5)	11 (26.2)
Plant-based diet pattern (Yes, N, %)	15 (32.6)	16(34.8)	2 (4.55)	13 (31.0)
Traditional Korean diet pattern (Yes, N, %)	21 (45.7)	18 (39.1)	26 (59.1)	18 (42.9)
Energy intake	1593 ± 59.8	1517 ± 61.1	1542 ± 61.1	1461 ± 61.9
EER %	61.2 ± 2.01	62.3 ± 2.0	57.8 ± 1.92	60.6 ± 2.06
CHO intake (En%)	54.1 ± 1.13	53.3 ± 1.15	53.4 ± 0.01	54.7 ± 0.01
Protein intake (En%)	15.9 ± 0.41	16.5 ± 0.42	16.4 ± 0.36	16.1 ± 0.36
Fat intake (En%)	29.3 ± 0.89	29.1 ± 0.93	28.9 ± 0.87	29.2 ± 0.28
Dietary fiber (g/day)	16.0 ± 0.73	15.8 ± 0.74	15.7 ± 0.54	16.1 ± 0.55
Kimchi (g/day)	76.9 ± 8.86	74.6 ± 9.05	82.3 ± 9.14	58.0 ± 9.24
Sugar beverage (g/day)	46.7 ± 9.70	32.8 ± 9.90	29.4 ± 10.4	33.4 ± 10.5
Beans (g/day)	20.4 ± 7.9	32.4 ± 8.03	16.2 ± 4.04	24.5 ± 4.09
Jang (g/day)	0.32 ± 0.20	0.18 ± 0.21	0.20 ± 0.26	0.55 ± 0.25
Chungkookjang (N, %)	-	-	23 (48.9)	21 (50.0)
Fruits (g/day)	77.3 ± 11.1	80.9 ± 11.3	73.0 ± 10.8	76.7 ± 11.0
Green vegetables(g/day)	26.6 ± 7.63	33.9 ± 7.79	16.4 ± 3.94	19.2 ± 3.99
White vegetables (g/day)	29.3 ± 5.56	29.0 ± 5.68	37.4 ± 5.08	32.1 ± 5.87
Mushroom (g/day)	1.70 ± 0.81	0.80 ± 0.81	4.26 ± 1.78	3.18 ± 1.80
Pickle (g/day)	2.20 ± 1.14	4.90 ± 1.17	2.96 ± 1.10	2.39 ± 1.12
Smoking (each cigarette/day)	0.89 ± 0.28	0.45 ± 0.28	1.13 ± 0.28	0.23 ± 0.28
Alcohol intake (g/day)	2.24 ± 0.43	1.67 ± 0.79	2.02 ± 0.44	1.76 ± 0.44

Values represent mean ± standard deviation or number (%). Covariates: age, gender, energy intake, smoke duration, alcohol drink amount, disease states, chungkookjang intake, exercise, and dietary fiber. Healthy diet pattern: grains, fish, and potato; plant-based diet: sweet bread, fruits, nuts, and mushrooms; traditional Korean diet, rice, soup, and kimchi. EER, estimated energy requirement.

## Data Availability

All data supporting this manuscript are provided in the Supplementary File.

## Supplementary Materials

The following supporting information can be downloaded at: https://www.mdpi.com/article/10.3390/nu17010138/s1, Table S1: Biochemical parameters between the CKJ and placebo groups at the baseline and final assessments.
