# Supplementary material for: Association of Mucin-Degrading Gut Microbiota and Dietary Patterns with Colonic Transit Time in Constipation: A Secondary Analysis of a Randomized Clinical Trial
Source: Nutrients. 2024 Dec 31;17(1):138. doi: 10.3390/nu17010138 (PMC11722837; doi:10.3390/nu17010138)
Supplement: Supplementary file 1 [file nutrients-17-00138-s001.zip › nutrients-3381178-supplementary.pdf]

Supplementary Table S1. Biochemical parameters between the CKJ and placebo groups at the baseline and final assessments

Parameter	CKJ (n=50) Baseline	Placebo (n=50) Baseline	p-value²	CKJ (n=46) 8 Weeks	Placebo (n=48) 8 Weeks	p-value²	Change CKJ	Change Placebo	p-value¹ CKJ	p-value¹ Placebo
WBC	5.35±1.15	5.24±1.43	0.6727	5.17±0.93	4.88±1.07	0.3734	-0.18±0.83	-0.35±1.08	0.1309	0.025
RBC	4.42±0.44	4.35±0.36	0.3621	4.36±0.43	4.36±0.37	0.1037	-0.06±0.18	0.01±0.24	0.0282	0.7068
Hemoglobin	13.24±1.21	12.99±1.37	0.3319	13.11±1.38	13.02±1.46	0.2248	-0.13±0.56	0.03±0.74	0.1142	0.7597
Hematocrit	40.08±3.26	39.18±3.60	0.1918	39.57±3.48	39.23±3.48	0.1392	-0.51±1.72	0.06±2.04	0.0426	0.8467
Platelet	251±53.6	260±43.1	0.3413	253±53.1	264±44.9	0.7735	1.78±27.8	3.24±22.5	0.6531	0.313
Na	139±1.70	139±1.96	0.8704	140±1.71	139±1.84	0.6273	0.40±1.77	0.22±1.92	0.117	0.4217
K	4.24±0.26	4.20±0.29	0.4494	4.18±0.28	4.18±0.25	0.5622	-0.06±0.32	-0.03±0.26	0.1903	0.487
CI	106±1.86	106±1.97	0.6021	106±2.00	106±2.07	0.6514	0.10±2.08	-0.10±2.32	0.7356	0.7622
ALP	57.80±14.79	54.20±15.74	0.2415	57.88±15.68	55.34±14.54	0.4189	0.08±6.76	1.14±6.29	0.9336	0.2062
GGT	15.24±11.54	14.16±9.95	0.6174	13.80±12.20	13.78±12.95	0.3566	-1.44±5.24	-0.38±6.16	0.0578	0.6648
AST	23.08±7.92	20.56±3.82	0.0465	22.84±5.06	21.74±4.91	0.2332	-0.24±7.26	1.18±4.15	0.816	0.0498

ALT	20.60±10.47	16.88±5.27	0.0279	19.74±9.05	18.04±6.76	0.0614	-0.86±6.46	1.16±3.86	0.3513	0.0387
Total bilirubin	0.81±0.33	0.88±0.34	0.2445	0.79±0.28	0.82±0.24	0.3926	-0.01±0.27	-0.06±0.28	0.7317	0.1358
Total protein	7.09±0.34	7.01±0.28	0.1868	7.03±0.32	7.06±0.39	0.0861	-0.06±0.31	0.05±0.33	0.1752	0.2833
Albumin	4.52±0.20	4.47±0.18	0.1676	4.50±0.21	4.51±0.22	0.0802	-0.03±0.18	0.04±0.22	0.2787	0.1715
BUN	13.74±3.09	12.46±3.96	0.0746	13.62±2.71	13.24±3.99	0.1779	-0.12±2.90	0.78±3.68	0.7714	0.1406
Creatinine	0.70±0.12	0.70±0.12	0.9265	0.68±0.12	0.70±0.12	0.1681	-0.01±0.06	0.00±0.07	0.1082	0.6604
Ca	9.59±0.32	9.54±0.37	0.4847	9.49±0.35	9.52±0.42	0.2665	-0.09±0.31	-0.02±0.39	0.0358	0.7705
P	3.65±0.41	3.53±0.44	0.188	3.58±0.43	3.62±0.49	0.0646	-0.06±0.35	0.08±0.43	0.2055	0.1782
Total cholesterol	184±27.5	180±27.7	0.5183	183±31.7	182.7±24.4	0.4541	0.10±15.7	2.68±18.5	0.9643	0.3111
Triglyceride	120±67.6	115±55.6	0.6874	110±65.3	117±66.6	0.4137	-10.7±41.6	1.16±47.3	0.1227	0.8074
HDL-C	57.2±14.0	58.0±14.2	0.8055	57.3±15.6	57.0±12.4	0.9817	0.02±4.97	-0.96±4.66	0.959	0.3739
LDL-C	106±25.5	102±27.5	0.5355	106±28.5	103±24.4	0.6312	0.08±13.0	0.84±13.9	0.9511	0.6325
Glucose	88.7±8.71	86.3±9.34	0.1685	87.9±9.30	86.3±8.91	0.2951	-0.82±4.48	0.02±5.78	0.4295	0.9795

WBC, white blood cell; RBC, red blood cell; Hb, hemoglobin; Hct, hematocrit; PLT, platelet; ALP, alkaline phosphatase; GGT, gamma-glutamyl transferase; AST, aspartate transaminase; ALT, alanine transaminase; BUN, blood urea nitrogen; CK, creatine kinase; LD, lactate dehydrogenase; hs-CRP, high-sensitive C-reactive protein.

Values are presented as mean ± SD.

[1] Analyzed by Paired t-test

[2] Analyzed by Independent t-test
